# A MEMS-Based Co-Oscillating Electrochemical Vector Hydrophone

**DOI:** 10.3390/mi13010143

**Published:** 2022-01-17

**Authors:** Anxiang Zhong, Mingwei Chen, Yulan Lu, Jian Chen, Deyong Chen, Junbo Wang

**Affiliations:** 1 State Key Laboratory of Transducer Technology, Aerospace Information Research Institute, Chinese Academy of Sciences, Beijing 100190, China; zhonganxiang19@mails.ucas.ac.cn (A.Z.); chenmingwei19@mails.ucas.ac.cn (M.C.); luyl@aircas.ac.cn (Y.L.); chenjian@mail.ie.ac.cn (J.C.); 2 School of Electronic, Electrical and Communication Engineering, University of Chinese Academy of Sciences, Beijing 100049, China

**Keywords:** co-oscillating vector hydrophone, MEMS, electrochemical vibration sensor

## Abstract

Aiming at the development needs of low-frequency and high-sensitivity vector hydrophones, this paper has developed a micro-electro-mechanical system (MEMS) based co-oscillating electrochemical vector hydrophone. We obtained the optimized geometric parameters through simulation analysis of the diameter of the rubber membrane, the length of the flow channel and the diameter of the flow holes. Based on the simulation results, electrodes were fabricated using MEMS technology, and were then assembled and tested. Device characterization was conducted, where the sensitivity and bandwidth were quantified as 0.5–150 Hz, −187 dB ref. 1 V/μPa, respectively. Compared with a previously reported co-oscillating vector hydrophone, the co-oscillating vector hydrophone developed in this article featured a lower working frequency band.

## 1. Introduction

Vector hydrophones are sensors that can detect underwater acoustic signals, and have a wide range of applications in military target monitoring, underwater reconnaissance, fish detection, and oil and gas exploration [1]. Compared with traditional hydrophones, vector hydrophones can not only detect the sound pressure of scalar signals, but also vector signals such as displacement, velocity, and acceleration. At the same time, the vector hydrophone has significant advantages in overcoming the ambiguity of port/starboard discrimination problems, reducing isotropic noise, and miniaturizing the sonar system [2,3].

According to different structures, vector hydrophones can be divided into co-oscillating vector hydrophones and gradient vector hydrophones [4,5]. A co-oscillating vector hydrophone is equipped with an acceleration sensor or a vibration sensor within a rigid shell, which drives the embedded sensor to vibrate synchronously in response to acoustic signal under measurement [6]. A gradient vector hydrophone consists of several sound pressure hydrophones, where the measurement of the vibration velocity at the center point is realized by measuring the sound pressure of each point and then obtaining the sound pressure gradient of each axis [7]. Compared with the gradient vector hydrophone, the co-vibration vector hydrophone has the advantages of small size and light weight [8,9]. 

According to the different working principles of the vibration sensors used, co-oscillating vector hydrophones are mainly divided into piezoresistive vector hydrophones, piezoelectric vector hydrophones, and capacitive vector hydrophones [10]. Among them, the piezoresistive type is simple in structure, but suffers from key limitations such as low sensitivity, high power consumption, and high thermal noise [11]. As a passive device, the piezoelectric type features low power consumption, low noise, and high sensitivity but poor static performance [12]. The capacitive structure has high sensitivity, but its installation angle is small and it is not suitable for use in complex marine environments [13,14].

At present, the effective frequency bands of the vector hydrophones mentioned are all above 10 Hz, and therefore they cannot effectively monitor low-frequency underwater acoustic signals [15]. Electrochemical vibration sensors are mainly composed of an electrolyte solution, sensitive electrodes, and two rubber membranes. Since the velocities of ion movements in the electrolyte solution are extremely low in comparison with electron velocities, electrochemical vibration sensors are inherently low-frequency devices [16,17]. However, the mainstream electrochemical vibration sensors are too large to be integrated into co-oscillating vector hydrophones, and the effective frequency bandwidth of the mainstream electrochemical vibration sensors cannot match the application requirements of vector hydrophones [18,19,20]. At present, vector hydrophones based on electrochemical vibration sensors have not been reported.

To solve this problem, we developed a vector hydrophone based on a MEMS electrochemical vibration sensor. The working bandwidth of the vibration sensor was optimized by adjusting electrode parameters to meet the low-frequency requirements of vector hydrophones. In addition, through the optimization of assembly structures, the overall size of the vibration sensor was effectively reduced to enable its inclusion in the vector hydrophone. 

## 2. Structure and Working Principle

**Figure 1:**
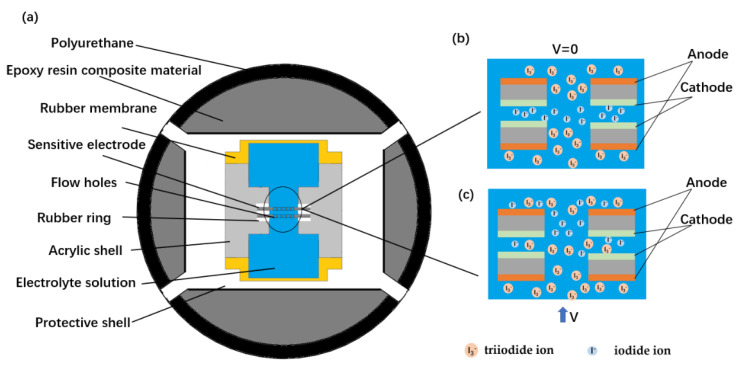
(**a**) Schematic diagram of the vector hydrophone based on the MEMS micro-electrochemical vibration sensor, consisting of: (1) an acrylic shell, (2) a pair of sensitive electrodes with many flow holes, (3) an electrolyte solution, (4) a rubber membrane, (5) a protective shell, (6) an epoxy resin composite material, and (7) polyurethane material. (**b**) Enlarged schematic diagram of the sensitive electrodes and the ion distribution in the absence of external vibration. (**c**) Enlarged schematic diagram of the sensitive electrodes and the ion distribution with an external vibration.

The structure diagram of the vector hydrophone based on the MEMS electrochemical vibration sensor is shown in Figure 1a, which mainly includes an internal MEMS electrochemical vibration sensor and external sealing materials. The MEMS electrochemical vibration sensor is the key part of the vector hydrophone, which mainly includes (1) an acrylic shell, (2) a pair of sensitive electrodes with many flow holes, (3) an electrolyte solution, and (4) two rubber membranes. The acrylic shell was used as a whole frame, and there was a compression channel with a diameter of 5 mm in the middle of the shell. Two pairs of sensitive electrodes were fixed in the middle of the flow channel, and were arranged according to the anode–cathode–cathode–anode setup. The two ends of the flow channel were pressed and sealed with rubber membranes to form a liquid storage cavity, and the mixed electrolyte solution of iodine and potassium iodide was filled in the flow channel and the liquid storage cavity. The external sealing materials mainly included three parts: a protective shell to protect the internal sensor, an epoxy resin composite to adjust the overall density of the vector hydrophone, and a waterproof material composed of polyurethane. 

When the co-oscillating vector hydrophone moves freely under water, if the size of the hydrophone satisfies the following relationship [3]:

(1) kL≪1

in which *k* is the wavelength, *L* is the maximum geometric size of the co-oscillating vector hydrophone, then there is the following relationship between the vibration velocity of the co-oscillating vector hydrophone and the vibration velocity of the water quality point at the center of the vector hydrophone in the sound field:

(2) $$V=\frac{3\rho_{0}}{2\bar{\rho }+\rho_{0}}V_{0}$$

in which *V* is the vibrational velocity of the co-oscillating vector hydrophone, $V_{0}$ is the vibration velocity of the water quality point at the center of the vector hydrophone in the sound field, $\rho_{0}$ is the density of water medium, and $\bar{\rho }$ is the density of the co-oscillating vector hydrophone.

Therefore, when the density of the co-oscillating vector hydrophone is the same as that of the water medium and the center of mass of the co-oscillating vector hydrophone coincides with the geometric center, the co-oscillating vector hydrophone can drive its internal vibration sensor to vibrate synchronously when it moves freely under water.

When the vector hydrophone based on the MEMS electrochemical vibration sensor is in operation, a DC voltage is applied between the anode and the cathode. The ions in the electrolyte solution redox on the surfaces of the anode and cathode [21,22,23]:

(3) $$\mathrm{Anode}:\;3-2e^{-}\to I_{3}^{-}$$

(4) $$\mathrm{Cathode}:\;I_{3}^{-}+2e^{-}\to 3I^{-}$$

A triiodide ion is produced near the anode and consumed near the cathode, while an iodide ion is produced near the cathode and consumed near the anode, leading to a difference in the concentration of the two ions between the electrodes. The two ions diffuse from high concentration to low concentration and finally reach dynamic equilibrium, as shown in Figure 1b [24]. 

In the absence of external vibration, due to the symmetrical placement of the two electrodes, the ion concentration in the electrolyte is symmetrically distributed. At this time, the output currents of the two cathodes are equal, and the output is zero after the circuit difference. In the case of an external vibration, since the two electrodes are fixed to the housing, the electrolyte moves relative to the electrodes due to inertia, which causes the ion concentrations on the electrode surface to change, as shown in Figure 1c [25]. At this point, the output current of the two cathodes is no longer equal, and the output is generated by the circuit difference [26].

## 3. Numerical Simulation

**Figure 2:**
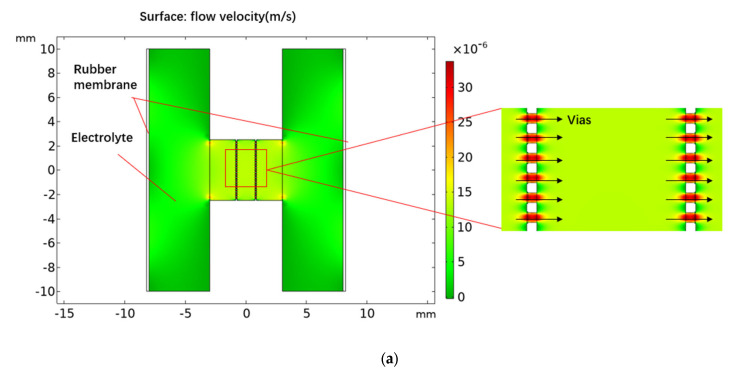

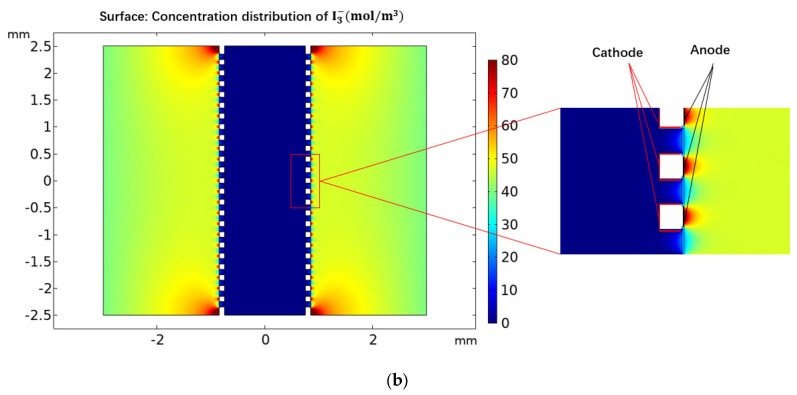
Simulation model of electrochemical vibration sensor: (**a**) simulation model of the vibration module; (**b**) simulation model of electromechanical module.

In order to study the characteristics of the MEMS-based co-oscillating electrochemical vector hydrophone, this study used finite element analysis (COMSOL Multiphysics 5.3) to simulate the sensor model. To simplify the analysis procedure, the simulation modules were divided into a vibration module and an electrochemical module, depending on different processes. The simulation model of the vibration module is shown in Figure 2a, in which the inputs of vibration velocities were converted into the outputs of electrolyte flow velocities at the entrance of the flow channel by parameter analysis based on the fluid–solid coupling physical field. The simulation model of the electrochemical module is shown in Figure 2b, which converted the electrolyte velocities into the current outputs on the electrodes based on the physical field of laminar flow and cubic current distribution.

In the simulation, key geometrical parameters including flow channel length, rubber membrane diameter, and flow hole diameter were fine-tuned. Among them, channel length and rubber membrane diameter were the main factors that affected the size of the electrochemical vibration sensor. By simulating the influences of channel length and rubber membrane diameter on the performance of the electrochemical vibration sensor, the device size was reduced under the condition of ensuring the performance of the electrochemical vibration sensor. The flow hole diameter mainly affected the flow resistance of the device, and the flow resistance could affect the effective frequency band, sensitivity, and other parameters of the device. Through the simulation of the flow hole diameter, the parameters of the flow hole diameter that met the requirements of the sensitivity and frequency band application of the vector hydrophone were determined for subsequent fabrications.

**Figure 3:**
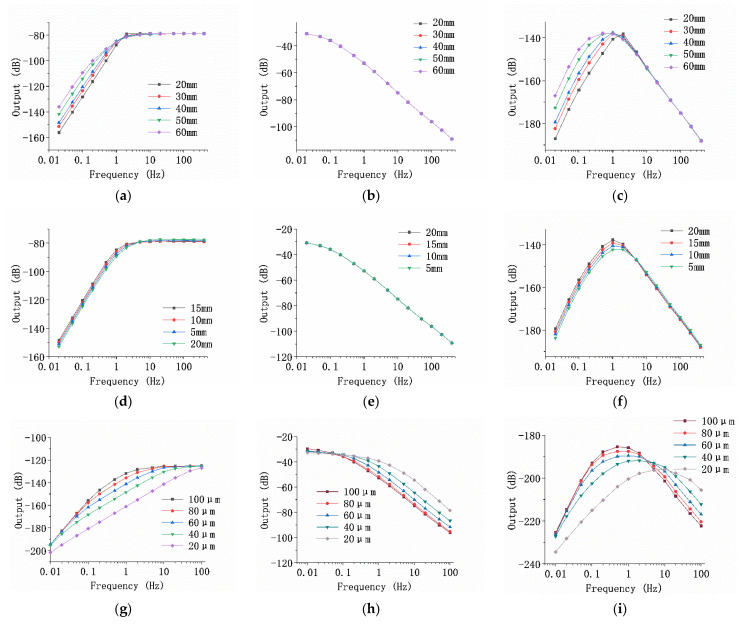
Simulation results of sensor optimization. (**a**) The amplitude–frequency curve of the simulation output of the vibration module when the diameter of the rubber membrane was 20–60 mm; (**b**) the amplitude–frequency curve of the simulation output of the electrochemical module when the diameter of the rubber membrane was 20–60 mm; (**c**) the amplitude–frequency curve of the overall output of the simulation model when the diameter of the rubber membrane was 20–60 mm; (**d**) the amplitude–frequency characteristic curve of the simulation output of the vibration module when the length of the flow channel was 5–20 mm; (**e**) the amplitude–frequency characteristic curve of the simulation output of the electrochemical module when the length of the flow channel was 5–20 mm; (**f**) the amplitude–frequency characteristic curve of the overall output of the simulation model when the flow channel length was 5–20 mm; (**g**) the amplitude–frequency curve of the simulation output of the vibration module when the diameter of the flow holes was 20–100 μm; (**h**) the amplitude–frequency characteristic curve of the simulation output of the electrochemical module when the diameter of the flow holes was 20–100 μm; (**i**) the amplitude–frequency characteristic curve of the overall output of the simulation model when the diameter of the flow holes was 20–100 μm.

Figure 3 shows the simulation outputs as a function of the diameter of the rubber membrane, the length of the flow channel, and the diameter of flow hole, respectively. The horizontal axis represents the frequency in Hz, and the vertical axis represents the output. On the whole, for the amplitude–frequency characteristic curve of the vibration module, the output increased at first and then did not change with the increase of frequency, and thus the amplitude–frequency characteristic of the vibration module indicated a high-pass link. For the electrochemical module, the response decreased with the increase of frequency, and the electrochemical module functioned as a low-pass link. With the comprehensive consideration of the vibration and electrochemical modules, the overall amplitude–frequency response first increased and then decreased with the increase of frequency. 

Figure 3a–c shows the simulation results of the effects of the diameter of the rubber membrane. When other factors remained the same, the diameter of the rubber membrane mainly affected low-frequency performance. The low-frequency attenuation speed decreased as the diameter of the rubber membrane increased, leading to better low-frequency performance. The reason was that the increasing diameter of the rubber membrane reduced its elastic coefficient, thus reducing the natural frequency of the sensor and improving the low-frequency performance.

Figure 3d–f shows the simulation results of the change of the channel length. When other factors remained the same, the change of the channel length slightly affected the low-frequency domain. The initial frequency point of low-frequency attenuation decreased as the channel length increased, and the attenuation rate was basically unchanged. The reason was that although the change of the channel length affected the inertia quality of the sensor, since the channel was narrow and the electrolyte was mainly located in the liquid storage chamber, the change of the channel length had little effect on the inertia mass of the sensor and the performance of the device.

Figure 3g–i shows the simulation results of the effects of the flow hole diameter. When other factors remained the same, the initial attenuation frequency of the vibration module became lower and the attenuation speed became faster with increasing flow hole diameter. On the whole, as the diameter of the flow holes decreased, the center frequency and the working bandwidth of the electrochemical vibration sensor increased, while the sensitivity of the sensor decreased. 

## 4. Fabrication

**Figure 4:**
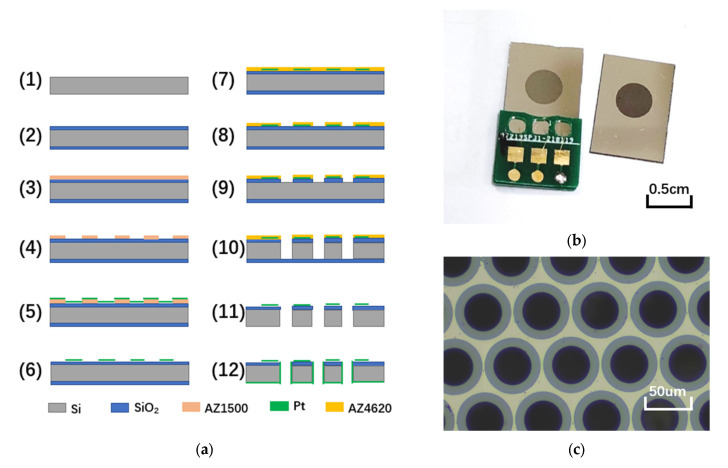
Manufacturing process of sensitive electrode. (**a**) Manufacturing process of sensitive electrode based on a micro-electro-mechanical system. (**b**) The finished electrode (right) and the electrode after pasting PCB (left). (**c**) Microscopic observation of flow holes in sensitive electrodes.

Micro-fabrication was conducted to manufacture sensitive electrodes as shown in Figure 4a. The sensitive electrodes were made of a 4 inch silicon wafer. The silicon wafer with a double-sided polishing thickness of 200 μm was cleaned, followed by thermal oxidation to form an insulating layer of 0.5 μm in thickness (Steps (1) and (2)). Then, the patterned metal platinum was obtained by electron-beam evaporation and lift-off was conducted to form anode electrodes (Steps (3) to (6)). The silicon dioxide layer on the surface of the flow holes was removed by reactive ion etching (RIE), and the silicon wafer was etched to form flow holes by deep reactive ion etching (DRIE) (Steps (7) to (10)). The residual silicon dioxide layer on the back of the flow holes was removed by RIE (Step (11)). Finally, the sidewall cathodes were formed by magnetron sputtering of metallic platinum (Step (12)).

**Figure 5:**
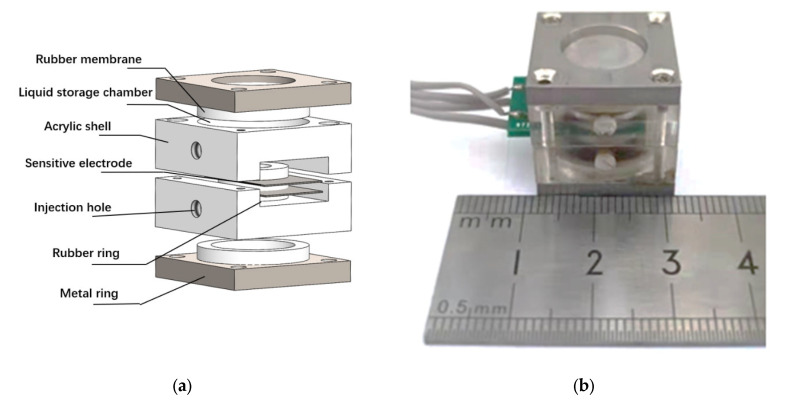
(**a**) The schematic diagram of electrochemical vibration sensor assembly. (**b**) The electrochemical vibration sensor after assembly.

The schematic of the sensor assembly is shown in Figure 5a. The fabricated sensitive electrodes were fixed between two acrylic shells by mechanical compression using a rubber ring, and then the rubber membranes were fixed on both sides of the acrylic shells by a metal ring. A liquid storage cavity was formed between the rubber membranes and the acrylic shells, with a liquid injection hole reserved on the side wall. Finally, the sensor assembly was completed by injecting the electrolyte through the injection hole, which was sealed afterwards. The assembled device is shown in Figure 5b, and the overall size of the sensor was 2 cm × 2 cm × 2 cm.

## 5. Experimental Characterization

**Figure 6:**
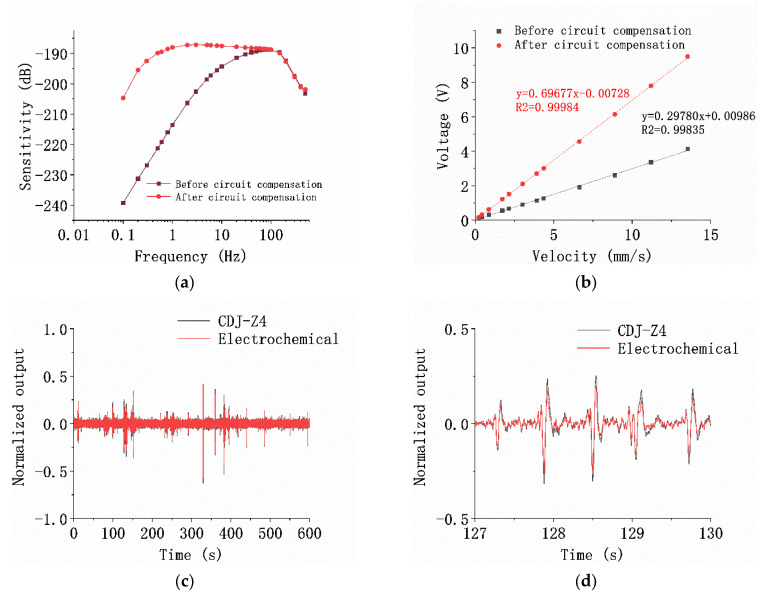
Test results of the sensitive structure of the MEMS electrochemical vector hydrophone. (**a**) Sensitivity curves of the device before (black curve) and after the circuit compensation (red curve); (**b**) linearity test results of the device at 10 Hz; (**c**) comparison test results between the electrochemical vector hydrophone sensitivity unit and the moving-coil detector CDJ-Z4; (**c**) the normalized time-domain response of the electrochemical vector hydrophone sensitivity unit and the moving-coil detector CDJ-Z4 are compared; (**d**) the enlarged view of the normalized time-domain response from 127 to 130 s.

In this study, a home-developed testing platform mainly composed of a shaking table [19] was adopted to characterize the fabricated MEMS-based co-oscillating electrochemical vector hydrophone. Figure 6a shows the sensitivity results, in which the abscissa represents the frequency in Hz and the ordinate indicates the sensitivity in dB. The center frequency, the effective frequency bandwidth, and the sensitivity for the sensor without circuit compensation [27] were quantified as 80 Hz, 20–150 Hz, and −188.86 dB ref. 1 V/μPa@80 Hz (563.44 V/(m/s)), respectively. After circuit compensation, the effective frequency band of the sensor was extended to 0.5–150 Hz, with a comparable sensitivity of −187.23 dB ref. 1 V/μPa (679.20 V/(m/s)).

Figure 6b shows the results of linearity tests at 10 Hz. The abscissa represents the input speed in mm/s and the ordinate indicates the output voltage in V. Under the condition that the total harmonic distortion of the output waveform was less than 3%, the input–output curve in the input speed range of 0.2 mm/s–13 mm/s was tested by adjusting the input speed of the shaking table. As for the results, the linear coefficients of the sensor with and without circuit compensation were quantified as 0.99835 and 0.99984, respectively.

Figure 6c shows the comparison of the time-domain consistency between the developed device and the commercial geophone CDJ-Z4 (Chongqing Geological Instrument Factory, Chongqing, China). The abscissa represents the time (in seconds), and the ordinate represents the normalized voltage. The natural vibration signal was tested for a period of time. The developed device was in good agreement with the commercial CDJ-Z4 geophone, and the corresponding correlation coefficient was 0.9742. Figure 6d is the enlarged view of the normalized time-domain response from 127 to 130 s.

As shown in Table 1, compared with the electrochemical vibration sensor in [19] and the commercial electrochemical vibrational sensor CME-6011 (R-sensors, Moscow, Russia), the electrochemical device developed in this paper had a higher working frequency band and smaller volume, but its sensitivity was lower.

Table 2 shows the working frequency band and sensitivity of the electrochemical vector hydrophone and of the vector hydrophones based on other principles. The electrochemical vector hydrophone had the lowest working frequency band and a comparable high sensitivity.

## 6. Conclusions

According to the requirements of vector hydrophones, this paper optimizes the size and frequency band of MEMS electrochemical vibration sensors to design a MEMS-based co-oscillating electrochemical vector hydrophone. The simulation results show that the size of rubber membrane and the length of flow channel had little effect on frequency above 1 Hz. However, the diameter of the flow hole had a great influence on the performance of the device. With the decrease of the diameter of the flow hole, the central frequency of the device increased, the effective frequency band of the device increased, but the sensitivity decreased. According to the test results, the size of the electrochemical vibration sensor was reduced from 8 × 8 × 6 cm^3^ to 2 × 2 × 2 cm^3^, the bandwidth of the MEMS based co-oscillating electrochemical vector hydrophone was 0.5–150 Hz, and the sensitivity in this frequency band was −187 dB ref. 1 V/μPa (679 V/(m/s)).

## Figures and Tables

**Table 1:** The effective frequency bands and sensitivities of co-oscillating vector hydrophones based on different working principles.

Device	Effective Frequency Band (Hz)	Sensitivity (V/(m/s))	Volume (mm^3^)
This paper	0.5–150	679	20 × 20 × 20
[19]	0.0083–20	2000	Φ80 × 60
CME-6011	0.033–50	2000	Φ204 × 210

**Table 2:** The effective frequency bands and sensitivities of co-oscillating vector hydrophones based on different working principles.

Working Principle	Effective Frequency Band (Hz)	Sensitivity (dB ref. 1 V/μPa)
Piezoresistive	20–1250	−185
Piezoelectric	20–2000	−191
Capacitive	20–1000	−190
Electrochemical	0.5–150	−187

## References

[1] Chen L.J., Yang S.E. (2006). A design of novel piezoresistive vector hydrophone. Appl. Acoust..

[2] Sun G.Q., Li Q.H. (2004). Progress of study on acoustic vector sensor. Acta Acoust..

[3] Chen H.J., Yang S.E. (2005). Design of co-vibrating vector transducer. Tech. Acoust..

[4] Tian R.Z., Chen B. (2009). Design of three-dimensional co-vibrating vector hydrophone. Acoust. Electron. Eng..

[5] Lv W.L., Pang M. (2010). Study on Optic Fiber Gradient Hydrophone Based on Composite Structures of Compliantly Variable Cylinder and Diaphragm. Acta Opt. Sin..

[6] Chen H.J., Hong L.J. (2003). Design of resonant type vector hydrophone based on piezoelectric accelerometer. J. Transducer Technol..

[7] Zhang T., Hu B.J. (2020). Design and Experiment of Three-dimensional Gradient Fiber Optic Vector Hydrophone. Semicond. Optoelectron..

[8] Jia Z.F. (2001). Design and characteristics of a resonant-sphere type three-dimensional vector hydrophone. Appl. Acoust..

[9] Jia Z.F. (1996). On pressure gradient hydrophone with co-oscillating sphere. Appl. Acoust..

[10] Li J.H., Ma J. (2018). Research progress of MEMS piezoelectric hydrophone and vector hydrophone. Appl. Acoust..

[11] Qiao H., Liu J. (2008). Design of a novel Si based bionic vector hydrophone based on piezoresistive effect. Chin. J. Sens. Actuators.

[12] Yang S.H., Ding J.W. (2018). Cross-Sector Ciliated MEMS Vector Hydrophone with High Sensitivity. Piezoelectrics Acoustooptics.

[13] Zhang Y.L., Gui C.Y. (2018). Research on silicon micro capacitive one-dimensional vector hydrophone. Appl. IC.

[14] Cao X.P., Zhang D.C. (2002). A triaxial differential capacitive silicon miniature accelerometer with large height. Chin. J. Sci. Instrum..

[15] Chen L.J., Zhang P. (2006). Overview of vector hydrophone. Transducer Microsyst. Technol..

[16] Krishtop V.G., Agafonov V.M. (2012). Technological principles of motion parameter transducers based on mass and charge transport in electrochemical microsystems. Russ. J. Electrochem..

[17] He W.T., Chen D.Y. (2012). Low frequency electrochemical accelerometer with low noise based on MEMS. Key Eng. Mater..

[18] Su R.T., Li W. (2014). Simulation of four-electrode electrochemical MEMS acceleration sensor. Transducer Microsyst. Technol..

[19] Xu C., Wang J.B. (2020). The electrochemical seismometer based on fine-tune sensing electrodes for undersea exploration. IEEE Sens. J..

[20] Qi W.J., Xu C. (2021). MEMS-based electrochemical seismometer with a sensing unit integrating four electrodes. Micromachines.

[21] Hurd R.M., Jordan W.H. (1960). The Principles of the Solion. Platin. Met. Rev..

[22] Sun Z.Y., Agafonova V.M. (2012). Numerical modeling of a four-electrode electrochemical accelerometer based on natural convection: The boussinesq flow model vs. The compressible flow model. Russ. J. Electrochem..

[23] Newson J.D., Riddiford A.C. (1961). The kinetics of the iodine redox process at platinum electrodes. J. J. Electrochem. Soc..

[24] Kozlov V.A., Terent’ev D.A. (2002). Frequency Characteristics of a Spatially-Confined Electrochemical Cell under Conditions of Convective Diffusion. J. Russ. J. Electrochem..

[25] Kozlov V.A., Safonov M.V. (2002). Dynamic Characteristic of an Electrochemical Cell with Gauze Electrodes in Convective Diffusion Conditions. J. Russ. J. Electrochem..

[26] Hai H., Agafonova V.M. (2013). Molecular Electric Transducers as Motion Sensors: A Review. Sensors.

[27] Zhang Z.Y., Wang J.B. (2015). Study of Bandwidth Expansion Based on Electrochemical Vibration Sensor. Key Eng. Mater..

